# Thermal hyperalgesia and dynamic weight bearing share similar recovery dynamics in a sciatic nerve entrapment injury model

**DOI:** 10.1016/j.ynpai.2021.100079

**Published:** 2021-12-06

**Authors:** Garrett D. Sheehan, Molly K. Martin, Violet A. Young, Rasheen Powell, Arin Bhattacharjee

**Affiliations:** a Program in Neuroscience, University at Buffalo, The State University of New York, Buffalo, NY 14203, USA; b Department of Pharmacology and Toxicology, University at Buffalo, The State University of New York, Buffalo, NY 14203, USA

**Keywords:** Ongoing pain, Nerve compression, Pain behavior

## Abstract

Chronic constriction injuries (CCI) of the sciatic nerve are widely used nerve entrapment animal models of neuropathic pain. Two common pain behaviors observed following CCI are thermal hyperalgesia and mechanical allodynia, measured by the Hargreaves and von Frey tests, respectively. While thermal hyperalgesia tends to recover by 30 days, mechanical allodynia can persist for many more months thereafter. Consequently, mechanical allodynia has been used extensively as a measure of ‘chronic pain’ focusing on the circuitry changes that occur within the spinal cord. Here, using the sciatic nerve cuff variant of CCI in mice, we propose that in contrast to these evoked measures of nociceptive hypersensitivity, dynamic weight bearing provides a more clinically relevant behavioral measure for ongoing pain during nerve injury. We found that the effect of sciatic nerve cuff on the ratio of weight bearing by the injured relative to uninjured hindlimbs more closely resembled that of thermal hyperalgesia, following a trend toward recovery by 30 days. We also found an increase in the percent of body weight bearing by the contralateral paw that is not seen in the previously tested behaviors. These results demonstrate that dynamic weight bearing is a reliable measure of non-evoked neuropathic pain and suggest that thermal hyperalgesia, rather than mechanical allodynia, provides a proxy measure for nerve entrapment-induced ongoing pain.

## Highlights

• The sciatic nerve cuff model of neuropathic pain exhibits pain recovery.
• Thermal hyperalgesia and dynamic weight bearing display similar pain recovery profiles, whereas mechanical allodynia persists.
• Dynamic weight bearing is a non-reflexive, pain assessment of ongoing pain during nerve entrapment.

## Introduction

Neuropathic pain is quickly becoming a global health issue (Cavalli et al., 2019), affecting the quality of life and the ability of those suffering to perform everyday tasks (Campbell and Meyer, 2006). Neuropathic pain is caused by damage to peripheral or central neuronal tissue of the somatosensory system (NIH HEAL Initiative 2021 *Annual Report*, 2021). As the burden of neuropathic pain increases, so too does the need for effective therapeutics (Kohrt et al., 2018, Mansfield et al., 2016). Accordingly, a number of animal models of peripheral neuropathic pain have been developed in order to understand the various cellular, molecular, and central circuit changes that occur as a result of peripheral nerve damage (Barrot, 2012). Most of these models induce irreversible damage to the sciatic nerve, and each provides unique features that help to understand the various mechanisms underlying neuropathic pain. For example, sciatic nerve crush studies have revealed a contribution of peripheral Wallerian degeneration and TRPV1 channels to the thermal hyperalgesia characteristic of this model (Ren et al., 2015), whereas in variations of the spared nerve injury (SNI) model, where the common peroneal and tibial nerve are cut, leaving the sural nerve intact, the contribution of central circuit changes to mechanical allodynia was uncovered (Decosterd and Woolf, 2000). With the variety of peripheral neuropathic pain models comes a variety of phenotypic consequences (Jaggi et al., 2011). The behavioral outcomes of sciatic nerve ligation (SNL) and SNI have been shown to differ considerably (De Vry et al., 2004, Decosterd and Woolf, 2000). Additionally, models such as SNL are subject to variability from differences in tightness of the ligatures placed on the nerve while variations in the selection of the spared nerve in SNI have been used (Bourquin et al., 2006, Langford and Mogil, 2008, Ro and Jacobs, 1993). The sciatic nerve cuff has been proposed as an alternative model providing more consistency between experimenters (Benbouzid et al., 2008, Yalcin et al., 2014). The sciatic nerve cuff model of neuropathic pain mimics common compression injuries and conditions like carpal tunnel, Morton’s neuroma, and sciatica (Padua et al., 2016, Ropper and Zafonte, 2015). Sciatic nerve cuffing faithfully results in mechanical allodynia persisting up to 100 days and thermal hyperalgesia which tends to recover within 30 days (Benbouzid et al., 2008, Tomasello et al., 2017, Yalcin et al., 2014). Thermal hyperalgesia and mechanical allodynia are typically measured by two reflexive or evoked assays, known as Hargreaves and von Frey, respectively (Bonin et al., 2014, Chaplan et al., 1994, Hargreaves et al., 1988, Pitcher et al., 1999). These evoked behavioral measures have been suggested to lack the ability to measure the ongoing, subjective experience of pain as it relates to chronic neuropathic pain (He et al., 2012, Huang et al., 2019). Dynamic weight bearing provides a means of measuring the behavioral outcome of chronic pain without relying on evoked measures (Quadros et al., 2015). In order to assess dynamic weight bearing, a mouse is simply placed in a plexiglass chamber, within which it is able to freely move about. During acquisition the weight applied to sensors lining the floor is measured. Through combining the sensor activation data with video data from above the animal, it is possible to assess the weight distribution changes of the subject as it relates to the forelimbs and hindlimbs. Time spent on limbs can also be measured. This assay has been used to study arthritic and inflammatory pain states (Gross et al., 2020, Kaneva et al., 2021, Powell et al., 2021). In this study we aimed to assess the impact of sciatic nerve cuff on dynamic weight bearing of the hindlimbs. We found an altered distribution of bodyweight that shifted toward the uninjured side, an effect whose recovery was remarkably paralleled by the recovery seen for thermal hyperalgesia. Here we propose that dynamic weight bearing provides a useful measure of ongoing pain in the sciatic nerve cuff model of neuropathic pain.

## Methods

### Animals

All animal experiments in this study were approved by the State University of New York at Buffalo Institutional Animal Care and Use Committee (IACUC) and adhered to the guidelines set forth by the National Institute of Health (NIH). A total of 16 C57BL/6 mice (8 males and 8 females) were used in this study, all purchased at 8 weeks of age from Envigo (Indianapolis, IN). Mice were housed individually on a 12-hour light dark cycle with food and water available *ad libitum*. All experiments were performed near the onset of the light cycle. Sample size was determined based on similar, previously published reports. We note all behavioral experiments were undertaken by a pair of investigators, one male and one female. The role of the experimenters was divided equally so that the exposure of the mice to each experimenter was exactly the same. The behavioral experiments were undertaken with two cohorts so that all data acquired on each baseline and post-surgery day was obtained by both the male and female experimenter. We include this information as gender of the experimenter has been shown to have sex-dependent effects on pain behaviors in rodents (Sorge et al., 2014).

### Nerve cuff model of neuropathic pain

For this study we turned to a common, well-established neuropathic pain model; the sciatic nerve cuff (Yalcin et al., 2014). Briefly, following six days of baseline thermal, mechanical, and dynamic weight bearing behavior, mice were placed under isoflurane anesthesia during which time a small 2–4 mm incision was made parallel to the femur of the right hind limb. The underlying muscle was then teased apart using wooden toothpicks exposing the sciatic nerve. A 2 mm PE-20 polyethylene tube was placed around the sciatic nerve and securely fastened so that the tube could rotate around the nerve but not come off. The sciatic nerve was then placed back into position, and the muscle was teased back together. The incision was then closed using 2 wound clips.

### Mechanical allodynia

In order to assess the mechanical sensitivity of the hind paws, we utilized the Simplified Up-Down Method (SUDO) (Bonin et al., 2014) with Touch Test Sensory Probes (Stoeltings, Wood Dale, IL). In brief, following a 15-minute habituation to the testing room, mice were placed in testing chambers on a raised platform with a metal wire mesh floor. After an additional 15-minute habituation to the testing chamber a probe of filament strength 0.16 g was applied to the plantar surface of the hind paw and the response was recorded as negative or positive. A positive response resulted in a smaller filament size on the subsequent application and a negative response resulted in the next largest filament application for a total of five applications per paw. Applications were spaced at a minimum of five minutes. The withdrawal threshold was then calculated with an adjustment factor based on the final presentation as in Bonin 2014.

### Dynamic weight bearing

On testing days, mice were habituated to the testing room for 30 min. Following habituation, mice were individually placed in the Dynamic Weight Bearing (DWB) Apparatus from Bioseb (France). The DWB apparatus was surrounded by a red plexiglass barrier in order to create a uniform environment within the clear testing apparatus. An initial 3-minute latency was followed by a 5-minute acquisition wherein the activation of pressurized sensors on the floor of the testing chamber was recorded in conjunction with video of the mouse from above. For each video, a minute and a half was later manually validated from the 5-minute acquisition at random, assigning the identity of the rear or front, left or right paws to activated pixels representing the pressure applied by each paw. After each acquisition, the mouse was then placed in another testing chamber to undergo thermal sensitivity testing.

### Thermal hyperalgesia

Thermal responsiveness was measured using an automated Hargreaves Apparatus (Ugo Basile, Italy). On the days where thermal responses were measured, mice were habituated in the room for 15 min, underwent DWB testing and then transferred to a plexiglass chamber placed on an elevated platform under which the Hargreaves Apparatus was maneuvered to reach the plantar surface of the hind paw. Latency to withdrawal was measured four times with a minimum five-minute latency between acquisitions and the average of four measurements was taken.

## Results

### Sciatic nerve cuff induces characteristic behavioral and anatomical changes

**Fig. 1:**
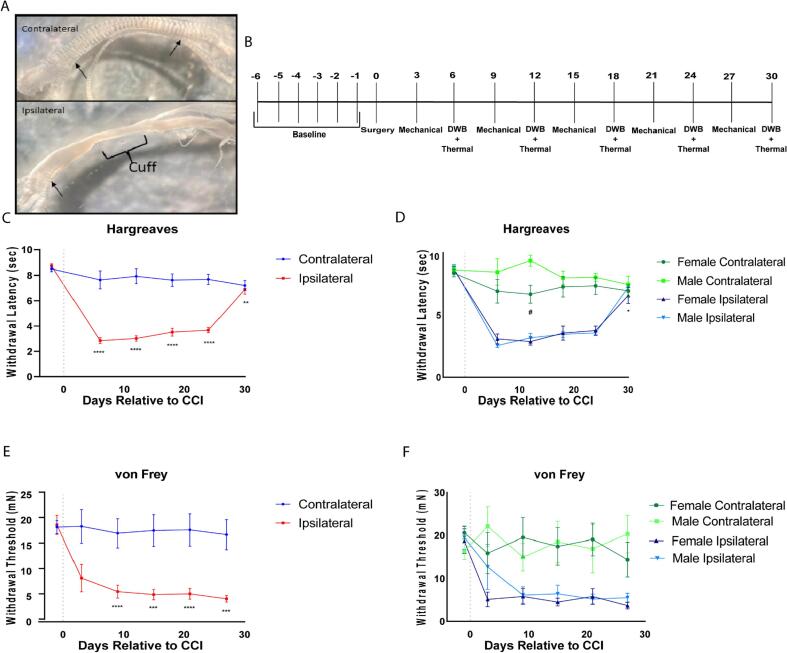
Behavioral and morphological consequences of sciatic nerve cuff. Morphological changes induced sciatic nerve cuff 10 days following placement. Bracket refers to placement of the cuff prior to removal. Arrows denote bands of Fontana (A). Experimental timeline (B). Sciatic nerve cuff induced a significant reduction in paw withdrawal latency as measured with Hargreaves apparatus on the ipsilateral, but not contralateral hind paw relative to baseline on all days following placement of the sciatic nerve cuff (n = 13). Baseline values were taken as the average paw withdrawal latency of three days prior to cuff placement (C). No sex differences were observed on the ipsilateral paw after cuff however there was a statistically significant difference between contralateral latencies of males and females on day 12 (#) (n = 6 males, n = 7 females). When taken separately, female ipsilateral paw withdrawal latency recovered to within baseline levels, however male ipsilateral paw withdrawal thresholds were significantly lower than baseline (*p = 0.0428) (D). Cuff placement induced a reduction in paw withdrawal threshold at days 9, 15, 21, and 27 on the ipsilateral but not for the contralateral paw as measured with von Frey filaments (E). No sex differences were observed for males vs. females for the ipsilateral paw withdrawal threshold (F). Significance was determined using two-way ANOVA with Bonferroni correction; ns: p > 0.05, *: p < 0.05, **: p < 0.005, ***: p < 0.0005, ****: p < 0.0001 relative to baseline, ns: p > 0.05, #: p < 0.05, ##: p < 0.005, ###: p < 0.0005, ####: p < 0.0001 for male vs female. Data is represented as mean paw withdrawal latency (sec) or threshold (mN) ± SEM. Multiple comparisons were performed for the ipsilateral and contralateral paws relative to baseline (C,E) and for paw by sex for each day (D,F).

The sciatic nerve showed morphological changes indicative of nerve compression 10 days after the cuff was placed. In Fig. 1A the contralateral (top) and ipsilateral (bottom) sciatic nerve are shown at 4.2× magnification. In the contralateral nerve, the bands of Fontana, the dark stripes that appear on the nerve, are visible and prominent. The bands of Fontana are thought to result from the undulating path taken by the axons within the sciatic nerve thus allowing for stretch of the nerve (Alvey et al., 2019). There is an apparent loss however of this banding on the cuffed ipsilateral nerve. This loss of banding has previously been shown to be due to inflammatory nerve compression, such as the compression provided from the cuff (Pourmand et al., 1994). The bracket labelled “cuff” indicates where the sciatic nerve cuff was positioned prior to removal following dissection of the sciatic nerve. Notably, the loss of the bands of Fontana extends beyond the area that would have been covered by the cuff. Additionally, there are visible bands at the end of the ipsilateral nerve, as indicated by the arrows indicating that the cuff-produced inflammation causes morphological changes dorsally and ventrally to the site of placement.

Initially we sought to test the utility of dynamic weight bearing in rodent models of neuropathic pain. Sixteen C57BL/6 mice (8 males and 8 females) were initially used for the behavioral part of the study. For rigor, at the end of the thirty-day-post-cuff experiment, all animals were sacrificed and dissected to ensure the presence of the sciatic nerve cuff and confirm its proper positioning for the duration of the trial. As a result, 2 males and 1 female were excluded from the analysis. All n-values represented are thus for n = 6 males, n = 7 females or n = 13 when pooled. Measurement of von Frey withdrawal thresholds were taken using the simplified up-down method on days 3, 9, 15, 21, and 27 following placements of the cuff (Bonin et al., 2014). On Days 6, 12, 18, 24, and 30, mice were first weighed, allowed to acclimate to the testing room for 30 min and then placed in the dynamic weight bearing apparatus for 7 min (5-minute acquisition with 2-minute latency). Immediately following measurement of dynamic weight bearing, the mouse was then allowed to acclimate for 15 min before beginning the testing of thermal hyperalgesia with a Hargreaves apparatus (Fig. 1B). This order was designed to minimize the impact of evoked behavioral measures on each other since the dynamic weight bearing acquisition allows for the animal to freely behave. For all statistical analyses, the testing days were compared to averaged values of three baseline testing days unless otherwise stated.

For Hargreaves testing, there was a significant (repeated measures two-way ANOVA with Bonferroni correction) reduction in the ipsilateral paw withdrawal latency relative to baseline (8.73 ± 0.16 sec) on days 6 (2.84 ± 0.23 sec, ****p < 0.0001), 12 (3.02 ± 0.22 sec, ****p < 0.0001), 18 (3.52 ± 0.31 sec, ****p < 0.0001), and 24 (3.68 ± 0.21 sec, ****p < 0.0001) following placement of the cuff (Fig. 1C). In line with previous reports the paw withdrawal latency showed a trend toward recovery by day 30 although it was still significantly reduced relative to baseline (6.89 ± 0.37 sec, **p < 0.005) (Benbouzid et al., 2008, Tomasello et al., 2017, Yalcin et al., 2014). There was no reduction in the paw withdrawal latency of the contralateral side relative to baseline (Fig. 1C). Comparison of males and females showed there were no sex differences for the ipsilateral paw withdrawal latency, however there was a statistically significant difference between male and female contralateral paw withdrawal latency on day 12 post cuff (female: 6.69 ± 0.73 sec, male 9.37 ± 0.45 sec, # p < 0.05) (Fig. 1D). Interestingly, when each sex was considered separately, female ipsilateral paw withdrawal latencies recovered by day 30 to a level not significantly different from baseline (6.59 ± 0.65 sec, p = 0.1563). This full recovery was not present in the males as the male ipsilateral paw withdrawal latency at day 30 still remained significantly different from baseline (7.25 ± 0.23 sec, *p = 0.0428).

Ipsilateral paw withdrawal thresholds to von Frey application were significantly reduced relative to baseline (19.07 ± 1.88 mN) beginning 9 days post-cuff (5.96 ± 1.27 mN, ****p < 0.0001), persisting through days 15 (5.40 ± 1.04 mN, ***p < 0.0005) and 21 (5.54 ± 1.08 mN ****p < 0.0001) to the end of the trial at day 27 (4.54 ± 0.66, ***p < 0.0005) (Fig. 1E). No sex differences were observed in the von Frey test (Fig. 1F).

Taken together, these results, in accordance with previous reports, illustrate the reliability of the sciatic nerve cuff in inducing pathophysiological and behavioral changes in mice.

### Sciatic nerve cuff induces changes in weight bearing

**Fig. 2:**
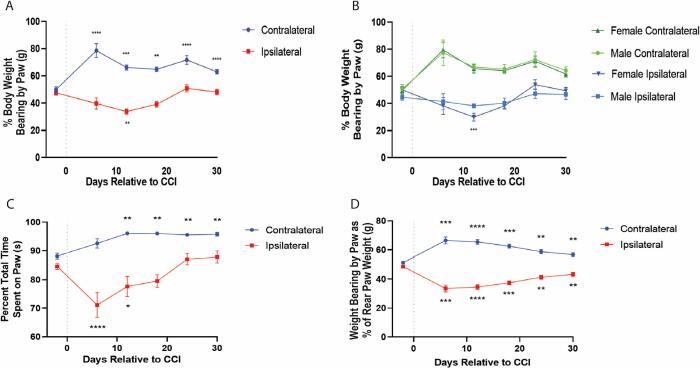
Changes in dynamic weight bearing following sciatic nerve cuff. Placement of the sciatic nerve cuff resulted in an increase in the percent of total body weight placed on the contralateral paw on all days after cuff placement as well as a decrease in the percent of body weight placed on the ipsilateral side on day 12 (n = 13) (A). When taken separately (n = 6 males, n = 7 females), only the females exhibited a decrease in the percent of body weight bearing by the ipsilateral paw on day 12 (***p < 0.0005) (B). When taken as the percent of total weight placed only on the rear paws, instead of total body weight, sciatic nerve cuff induced an ipsilateral decrease and a contralateral increase on all post-cuff days (C). The percent of total trial time that the mice placed weight on the ipsilateral paw decreased on days 6 and 12 post-cuff and increased contralaterally on days 12 through 30 (D). Significance was determined using two-way ANOVA with Bonferroni correction; ns: p > 0.05, *: p < 0.05, **: p < 0.005, ***: p < 0.0005, ****: p < 0.0001. Data is represented as mean percent total body or mean percent of total rear paw weight bearing, or mean percent total trial time that weight was placed on the paw ± SEM. Multiple comparisons were performed for the ipsilateral and contralateral paws relative to baseline (A) and for paw by sex for each day (B).

To assess the effect of sciatic nerve cuff on dynamic weight bearing, mice were tested in the dynamic weight bearing apparatus (BIOSEB) on the same days prior to thermal testing. When compared to baseline (50.05 ± 1.9%), all mice showed a significant increase in the percent body weight bearing by the contralateral side on days 6 (78.57 ± 5.12%, ****p < 0.0001), 12 (66.25 ± 1.86%, ***p < 0.0005), 18 (64.77 ± 1.63%, **p < 0.005), 24 (71.74 ± 3.25%, ****p < 0.0001) and 30 (62.93 ± 1.13%, ****p < 0.0001). Weight bearing by the ipsilateral paw was decreased on day 12 (33.78 ± 1.99%, **p < 0.005) relative to baseline (47.54 ± 1.73%) (Fig. 2aA). When separated by sex, only females exhibited a decreased ipsilateral paw withdrawal latency at day 12 (38.29 ± 1.26%, ***p < 0.0005) compared to baseline (44.61 ± 2.14%) (Fig. 2B). Further analysis revealed that when taken as a percentage of the weight placed on the sum of the rear paws, the mice exhibited a decrease in weight bearing by the ipsilateral paw on days 6 (48.73 ± 0.73%, ***p < 0.0005), 12 (34.58 ± 1.65%, ****p < 0.0001), 18 (37.47 ± 1.37%, ***p < 0.0005), 24 (41.27 ± 1.48%, **p < 0.005), and 30 (43.31 ± 1.38%, **p < 0.005), when compared to baseline (48.73 ± 0.73%). This decrease was mirrored by an increase in the percent of rear paw weight bearing by the contralateral side on days 6 (66.42 ± 2.40%, ***p < 0.0005), 12 (65.42 ± 1.65%, ****p < 0.0001), 18 (62.53 ± 1.37%, ***p < 0.0005), 24 (41.27 ± 1.48%, **p < 0.005), and 30 (56.69 ± 1.38%, **p < 0.005) when compared to baseline (51.27 ± 0.73%) (Fig. 2B). There were no sex differences observed when comparing weight bearing as a percent of total rear paw weight. In addition, the amount of time, taken as the percent of total trial time that the animal placed weight on the ipsilateral paw was decreased at days 6 (71.15 ± 4.38%, ****p < 0.0001) and 12 (77.61 ± 3.53%, *p < 0.05) when compared to baseline (84.64 ± 1.06%) (Fig. 2C). Time spent bearing weight by the ipsilateral paw returned to within baseline levels on days 18 (79.54 ± 2.19%), 24 (87.15 ± 1.93%), and 30 (87.87 ± 2.04%). Further, the amount of time that the mice placed weight on the contralateral paw increased on days 12 (96.08 ± 0.56%, **p < 0.005), 18 (96.05 ± 0.48%, **p < 0.005), 24 (95.57 ± 0.53%, *p < 0.05), and 30 (95.79 ± 0.65%, *p < 0.05) when compared to baseline (88.17 ± 1.02%) (Fig. 2D) There were no observable sex differences.

### Recovery of dynamic weight bearing following sciatic nerve cuff mirrors that of thermal paw withdrawal latency

**Fig. 3:**
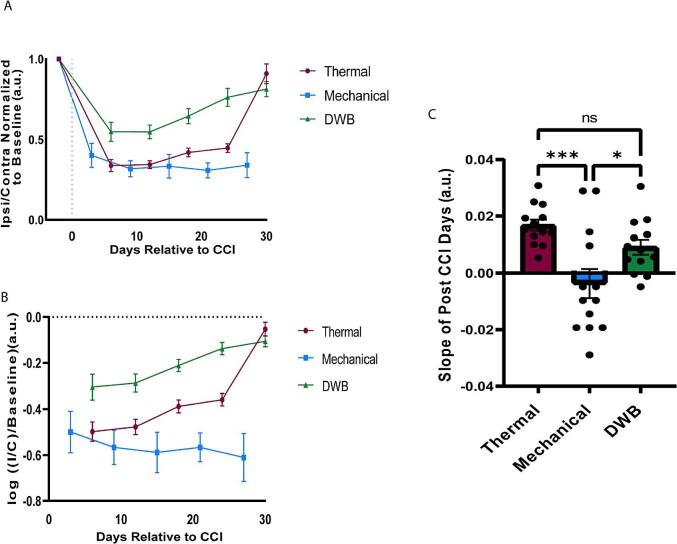
Thermal hyperalgesia and dynamic weight bearing exhibit recovery whereas mechanical allodynia persists. The ipsilateral to contralateral ratios of weight bearing, paw withdrawal latency, and paw withdrawal threshold were normalized to baseline and plotted in order to compare the three behavioral measures (A). For all days following surgery, the log of the ipsilateral to contralateral ratio normalized to baseline was plotted (B). The slope of the lines in (B) for each animal (n = 13) were compared in (C) and one-way ANOVA with Tukey post-hoc analysis was used to determine significance; ns: p > 0.05; *: p < 0.05; **: p < 0.005; ***: p < 0.0005; ****: p < 0.0001. All data points are represented as arbitrary units (a.u.).

In order to make a comparison between all three behavioral measures used in this study, we plotted the ipsilateral to contralateral ratio of paw withdrawal latency, paw withdrawal threshold, and percent body weight bearing for all animals normalized to the average of all three days of baseline behavior (Fig. 3A). In doing so, we noticed that the previously reported recovery in thermal paw withdrawal latency appeared to be mimicked by the ipsilateral to contralateral ratio plotted for percent body weight bearing in the dynamic weight bearing test (Fig. 3A) (Benbouzid et al., 2008, Tomasello et al., 2017, Yalcin et al., 2014). To explore this further, we calculated the log of the normalized ipsilateral to contralateral ratio for all behaviors and plotted them for all days following placement of the cuff (Fig. 3B). Throughout this time course the log of the normalized ipsilateral to contralateral ratio for thermal and dynamic weight bearing trended toward zero, indicating a ratio closer to one and thus a recovery towards baseline while the values for mechanical withdrawal threshold in the von Frey task maintains throughout the post-cuff days (Fig. 3B). In order to quantify this recovery, we calculated and plotted the slopes of these values for the individual animals in each task (Fig. 3C). The mean slope in arbitrary units for von Frey paw withdrawal thresholds (−0.004 ± 0.005) was significantly different from Hargreaves paw withdrawal latency (0.017 ± 0.002, ***p < 0.0005) and dynamic weight bearing (0.009 ± 0.002, *p < 0.05) as assessed by one-way ANOVA with Tukey post hoc analysis, while the slopes for Hargreaves and dynamic weight bearing were not significantly different (Fig. 3C). Taken together, these results suggest that there is a post-cuff recovery in the dynamic weight bearing behaviors that resembles thermal hyperalgesia recovery.

## Discussion

Here we have illustrated the reliability of the sciatic nerve cuff model of neuropathic pain in replicating previously reported behavioral and anatomical changes and have demonstrated the utility of dynamic weight bearing as an additional measure of neuropathic pain in mice. Our results revealed a previously unrecognized phenomenon in a parallel recoverability phenotype of thermal withdrawal latency as measured by the Hargreaves assay, and dynamic weight bearing alterations that follow sciatic nerve cuff injury.

As with the ipsilateral decreases seen in evoked withdrawal from thermal and mechanical stimuli (Fig. 1C,D), we expected to see an ipsilateral decrease in the percent of total body weight bearing by the ipsilateral paw. Indeed, there was a decrease in weight bearing by the ipsilateral paw seen on day 12, as well as time spent on the hind paw. Interestingly though, there was actually an increase in the weight bearing by the contralateral paw across each day following placement of the cuff. When plotted as the ratio of weight bearing by the ipsilateral paw relative to the contralateral paw there appeared to be a recovery, with the ratio trending toward baseline levels by 30 days following the initial post-cuff decrease (Fig. 3A). When we plotted the log of this ratio relative to baseline for each behavior for all days following placement of the cuff, we found that the slopes of the lines for all post-cuff days for dynamic weight bearing and thermal hyperalgesia trended toward zero, indicating a ratio of one, or a return to baseline and thus a “recovery” (Fig. 3B). In fact, when the slopes of these lines for each animal were considered, the mean values for the slopes after cuff placement for thermal and dynamic weight bearing were not statistically different, but both differed from von Frey sensitivity (Fig. 3C).

Mechanical allodynia has been shown to persist for up to 100 days following cuff and the recovery of thermal hyperalgesia by 30 days has been documented (Yalcin et al., 2014). The recovery of dynamic weight bearing within this window surprised us in that we anticipated it to follow von Frey sensitivity. Nerve injury models of neuropathic pain such as the SNI, SNL, and sciatic nerve cuff are thought to be mediated by several peripheral and central mechanisms. One such peripheral mechanism is plasticity of the primary afferent fibers resulting in alterations of their firing properties (Dib-Hajj et al., 1999, Study and Kral, 1996, Woolf and Salter, 2000). In SNI, central mechanisms are the most likely contributor to the mechanical allodynia observed in this model (Decosterd and Woolf, 2000) as two nerves are severed. When this is considered along with the absence of thermal hyperalgesia in SNI, it suggests that the thermal sensitivity observed in the cuff model is peripheral, rather than central in origin. In addition, the use of capsaicin patches, lidocaine patches, and nerve block as analgesic treatment for neuropathic pain indicates peripheral mechanisms are the driving force of clinical neuropathic pain (Backonja et al., 2008, Blair, 2018, Fujita, 1993, Pickering et al., 2019). As we consider the non-evoked nature of the dynamic weight bearing tool and the parallel recoverability phenotype it shares with thermal sensitivity, it is possible that the fibers mediating thermal sensation are those that generate the ongoing pain experienced in neuropathic models (He et al., 2012, Huang et al., 2019) and this ongoing pain leads to the non-evoked weight bearing changes observed in this model. Alternatively, it is possible that sciatic nerve cuff activates silent nociceptors (Jonas et al., 2020, Michaelis et al., 1996) which become mechanically sensitive following injury. Silent nociceptors have been shown to be sensitive to noxious thermal stimuli and insensitive to mechanical stimuli. However, following injury, these nociceptors then become sensitive to mechanical stimuli possibly due to a disinhibition of the mechanically sensitive ion channel Piezo2 (Prato et al., 2017). If these silent nociceptors do become activated following sciatic nerve cuff, then it is possible that their plasticity underlie the recoverable thermal phenotype and their acquisition of mechanical sensitivity could explain the impact on dynamic weight bearing. As mechanical allodynia has been shown to be mediated centrally in other neuropathic models such as SNI, this would explain the persistence of the mechanical allodynia despite the apparent recovery of the potentially silent nociceptor-mediated thermal hyperalgesia and dynamic weight bearing phenotype observed in this study. Moreover, von Frey filaments are incapable of activating these mechanosensitive silent nociceptors (Huang et al., 2019).

It is worth noting that the dominant effect of cuff on dynamic weight bearing was actually an increase in the percent of body weight bearing by the contralateral hindlimb. There was a decrease in weight bearing by the ipsilateral paw that reached significance at day 12 and returned to within baseline levels. It is possible that the contralateral increase represents a centrally mediated coping mechanism in response to the persistent input from the ongoing pain emanating from the injured limb. This would suggest that mechanical allodynia exists as a sensitizing mechanism allowing an organism to stay alert of previous damage and avoid subsequent injury. Indeed, mechanical allodynia returns to baseline in the cuff model when tested 100 days later (Benbouzid et al., 2008). The peripherally mediated ongoing pain, which coincides with thermal hyperalgesia, would recover as the injury subsides lest this pain continue and become maladaptive for the organism.

In conclusion, we believe that dynamic weight bearing provides a measure of ongoing neuropathic pain and thus represents a more clinically relevant pain measure over the traditionally used evoked measures. In fact, we were able to observe two salient behaviors using dynamic weight bearing: decreased weight bearing on the injured hindlimb, and increased weight bearing on the contralateral limb. These additional non-reflexive components should be able to further our understanding of neuropathic pain and aid in analgesic development. In addition, we believe that reflexive thermal hyperalgesia, as opposed to von Frey sensitivity, may be a more reliable measure of ongoing pain experienced by an animal following peripheral nerve compression.

## Declaration of Competing Interest

The authors declare that there is no conflict of interest regarding the publication of this paper.
